# A review on the impacts of COVID-19 on the auditory system: Implications for public health promotion research

**DOI:** 10.34172/hpp.2023.33

**Published:** 2023-12-16

**Authors:** Samin Shibafar, Fatemeh Jafarlou

**Affiliations:** ^1^Student Research Committee, Tabriz University of Medical Sciences, Tabriz, Iran; ^2^Department of Audiology, School of Rehabilitation Sciences, Tabriz University of Medical Sciences, Tabriz, Iran

**Keywords:** Conductive hearing loss, COVID-19, Hearing loss, Mixed hearing loss, Sensorineural hearing loss, Sudden hearing loss

## Abstract

**Background:**

Currently, there are few studies on the relationship between COVID-19 and the auditory system. In the current study, a review of the studies conducted in the fields of etiopathology, clinical manifestations, research, and treatment of hearing loss caused byCOVID-19 was conducted, which can be used as a baseline for future studies.

**Methods:**

We utilized the research approach suggested by Arksey and O’Malley to carry out this scoping review. Search was conducted in Farsi and English with a focus on the onset of hearing loss in patients with COVID-19 through Medline and PubMed, and Google Scholar search engine. Studies included were those involving adult patients diagnosed with COVID-19 who experienced hearing loss, ear pain, ear discharge, and otitis media. Studies were eligible for inclusion if there was a description of the otologic dysfunction, specifically onset, duration, or clinical outcomes.

**Results:**

Among 90 studies identified, 35 studies were included in the review process. Our findings suggest several possible mechanisms for sudden sensorineural hearing loss (SSNHL) in COVID-19 patients, and COVID-19 infection could have deleterious effects on the inner ear, specifically on the hair cells of the cochlea despite patients being asymptomatic and early identification of SSNHL in COVID-19patients can save the hearing and also patient.

**Conclusion:**

Hearing loss in COVID-19 infection has not received much attention by health care professionals. Sensorineural hearing loss (SNHL), tinnitus, and/or vertigo have been shown to occur during and following COVID-19 infection. Due to lack of research studies, and the inconsistency and even contradictory of the findings, it remains questionable whether COVID-19 contributes to the high incidence of hearing loss. The proper understanding of the mechanisms behind hearing loss in COVID-19 infections needs further research. However, it seems likely that SNHL could be included among the manifestations of those-called "long COVID" syndrome.

## Introduction

 In December 2019, the coronavirus pandemic (COVID-19) caused a worldwide health crisis, affecting millions of people. More than 5 million infections have been reported so far.^1-3^ According to research, COVID-19 has a lower death rate than MERS and SARS, however the rate statistics are diverse in different countries.^4^ COVID-19 is transmitted through airways and enters body cells by penetrating the angiotensin-converting enzyme 2 (ACE2) in the lungs.^5^

 ACE2 is a receptor found in various body tissues, including the lung, nose, heart, kidney, and intestine.^6^ Also, this enzyme is abundant in the brain and therefore in the temporal lobe. So, it is assumed this virus can easily affect the central auditory pathways.^7^ During penetration of the virus into the enzyme, by lowering the cytosolic pH, it becomes easier for the virus to bind to the ACE2 receptor; since cytosolic pH decreases with age, the virus easily causes a more severe infection in the elderly. The virus attaches to the beta chain of hemoglobin and penetrates red blood cells. So, it can be also transported with red blood cells and possibly infect all tissues with ACE2 receptors in their structure. There is a large amount of ACE2 in the tissues of the brain and medulla. ACE2 overexpression in the brain, except the medulla, has a positive effect on anti-inflammatory antioxidant and blood pressure regulator. However, if the cytosolic PH is low, the increase of ACE2 increases the viral load as well, and thus, the infection of COVID-19 may progress more vigorously.^5^ After entry, the virus spreads systemically and causes clinical symptoms of COVID-19.^6^

 In fact, SARS-CoV-2 is a large, enveloped positive-strand RNA virus, and the widespread infectivity of COVID-19 poses a health threat to the world entirely,^8^ The average incubation period of COVID-19 has been estimated to be 2 to 14 days after contact,^9^which has also been shown to be closely related to the immune system and the age of the patient.^4^ The common clinical manifestations of COVID-19 are fever, cough, fatigue, sore throat, headache, gastrointestinal symptoms, smell, and taste disturbances, which may turn into shortness of breath or, into more severe cases, SARS.^8,10^ However, COVID-19 is primarily a respiratory disease. The virus colonizes and multiplies in the respiratory system and involves the nasopharynx, trachea, bronchi, and then the lungs, which causes numerous symptoms. Afterwards, this virus affects many body organs such as digestive, nervous, musculoskeletal and kidney system.^11^ The virus may also affect the auditory system.

 Thrane et al, in a current cohort study on 225 COVID-19 patients with sudden sensory neural hearing loss found that more than 10% of the patients complained of hearing loss while more than 16% complained of tinnitus.^1^ They also claimed that the virus, like other viruses, can induce inflammatory effects in the end organs of the auditory system, including the organ of Corti and hair cells.^7^ Another study^12^ showed that the conductive element of the hearing pathway can be affected by ascending infections of the nasopharynx, which may lead to fluid accumulation in the middle ear. The initial manifestation of SARS-CoV-2 infection in the auditory system has been reported to be otalgia and tinnitus.^12^ Upper respiratory tract infections may often cause eustachian tube dysfunction due to mucosal swelling, which causes the symptoms such as conductive hearing loss (CHL), ear fullness/ear congestion, tinnitus, and vertigo.^1^ In terms of the potential type of hearing loss, previous case reports and case series studies^13-16^ mostly reported Sensorineural hearing loss (SNHL). This may be caused by the direct effect of SARS-CoV-2 on the organs of Corti, stria vascularis, and/or spiral ganglion. On the other hand, in some cases, CHL with otalgia and tinnitus has been reported, for instance one report described a 35-year-old woman displaying signs of a bulging eardrum and experiencing hearing impairment. In another study it was found that 75 percent of the participants had middle-ear effusion while 37.5% had otitis media.^12,17^ as mentioned above, hearing loss in COVID-19 infection has not received much attention by health care professionals. SNHL, tinnitus, and/or vertigo have been shown to occur during and following COVID-19 infection. Due to lack of research studies, and the inconsistency and even contradictory of the findings, it remains questionable whether COVID-19 contributes to the high incidence of hearing loss. The proper understanding of the mechanisms behind hearing loss in COVID-19 infections needs further research. However, it seems likely that SNHL could be included among the manifestations of those-called “long COVID” syndrome. The aim of this study was to comprehensively review the studies conducted in this field.

## Materials and Methods

###  Study design

 The methodological framework proposed by Arksey and O’Malley^18^and the PRISMA guideline^19^ was used to conduct this scoping review. For the purpose of understanding the ideas and evidence in a specific research area the preferred method was to conduct a scoping review. This approach involved identifying, collecting and summarizing literature that pertains to the topic at hand. By doing it aimed to uncover the concepts that form the foundation of this research field and determine the main sources and types of evidence that exist. The following five steps were used: (a) identifying a clear research objective and search strategies, (b) identifying relevant research articles, (c) selecting research articles, (d) extracting and charting data, and (e) summarizing, analyzing, discussing, and reporting the results.

###  Identifying the review questions

 The main review question was as follows: “What are the effects of COVID-19 on the auditory system?

###  Literature search strategies

 To conduct this study, a search was conducted in Farsi and English with a focus on the onset of hearing loss in patients with COVID-19 through Medline and PubMed databases (for each approach we utilized Mesh terms obtained from the PubMed database), and Google Scholar search engine from January 1, 2020 to May 2022. Also, the keywords of “Covid 19 and hearing loss” or “Covid 19 and conductive hearing loss”, or “Covid 19 and sensorineural hearing loss” or “Covid 19 and sudden hearing loss” or “Covid 19 and otitis media”, or “Covid 19 and inner or middle ear” were used. Apart, from conducting searches, on databases we also thoroughly examined the reference lists of the studies included and relevant reviews to identify any studies that could be included in this review.

 Two reviewers, FJ and SS conducted a review of the titles and abstracts of the studies found. They individually examined the text of the studies that passed the screening. In case FJ and SS had any differences, in their assessments they. Reached a consensus through dialogue.

###  Eligibility criteria

 We determined the criteria, for inclusion using the PICOs framework, which is a modified version of the PICO framework. The PICOs framework considers the population, phenomena of interest, context and study design.^20^ The following inclusion criteria were used: The population of interest was adult patients who were diagnosed with COVID-19 (SARS-CoV-2), using any diagnostic tool available for this disease (antigen test, antibody test, real-time polymerase chain reaction [RT-PCR]), and the patients experienced or diagnosed with hearing loss, ear pain, ear discharge, and/or otitis media. Studies were eligible for inclusion if there was a description of the otologic dysfunction, specifically onset, duration, and/or clinical outcomes. Randomized and nonrandomized controlled trials, and cohort, case-control, cross-sectional, case series, and case report studies were included. Studies involving the pediatric population were excluded. Inclusion criteria were established SARS-CoV-2 infection, cases of new onset hearing loss evaluated by audiological tests and temporal correlation between the two events. Exclusion criteria were non-confirmed SARS-CoV-2 infection and unclear temporal link between the two events.

###  Data extraction from included studies

 The data from all included studies were extracted by authors, and presented in Table 1. The data extracted for each article were authors’ name, location of study, sample size, study design, method and their measurements tools and outcomes. We employed Microsoft Excel, a software developed by Microsoft Corporation for organizing and synthesizing data.

**Table 1:** General characteristics of the reviewed articles

**Author**s	**Location**	**Participant (s)**	**Design**	**Method**	**Type of hearing loss**	**Outcomes**
Chern, et al (2021)^21^	New York	18 years	Case report	Tympanometry audiometry	Bilaterally SNHL	Bilateral SSNHL
Elibol (2020)^22^	Turkey	155	Retrospective	Medical files	SNHL	SSNHL 0.6%
Fidan (2020)^12^	Turkey	35	Case report	Tympanometry audiometry	CHL	Otitis media
Kilic et al (2020)^23^	Turkey	5	Case report	Audiometry	Unilateral SNHL	Unilateral SSNHL 20 %
Özçelik Korkmaz et al (2020)^24^	Turkey	116	Cohort	Questionnaire	SNHL	Hearing impairment 5.1%
Koumpa et al (2020)^15^	England	45 years	Case report	Audiometry	Unilateral SNHL	Hearing loss, tinnitus
Lamounier et al (2020)^10^	Brazil	67 years	Case report	Audiometry	SNHL	SSNHL
Lang et al (2020)^25^	Ireland	30 years	Case report	Audiometry, imaging	SNHL	SSNHL
Malayala and Raza (2020)^16^	USA	29 years	Case report		Hearing loss was not reported	Vestibular neuronitis
Miri and Ajalloueyan (2020)^26^	Iran	2	Case report		Hearing loss was not reported	Otalgia
Mustafa (2020)^27^	Egypt	20	Case-control	TEOAE VEMP	SNHL	Hearing loss 87.5 %
Raad, et al (2020)^17^	Iran	8	Case series	Otoscopy audiometry tympanometry	CHL	Otitis media 37.5 % Acute otitis media with perforation 12.5 %
Vanaparthy et al (2020)^28^	USA	63 years	Case report	Physical examination	Hearing loss was not reported	Vestibular neuritis
Parrino et al (2022)^29^	Italy	2761	ORS	audiometric test and videonystagmography	SNHL	34 (1.23%) SSNHL
Aslan et al (2021)^30^	Turkey	NR	OPS		Hearing loss was not reported	No relationship between COVID-19 and cases of SSNHL and Bell’s palsy was observed.
Yaseen et al (2021)^31^	Iraq	4850	ORS	Audiometry tympanometry	SNHL	SSNHL 26 (0.53%)
Chari et al (2021)^32^	Qatar	35 years	Case report	Audiometry tympanometry	SNHL	Unilateral SNHL
Gunay et al (2021)^33^	Turkey	23 years	Case report	Audiometry tympanometry	Mixed HL	Bilateral mixed hearing loss and ear pain
Abdel Rhman and Abdel Wahid (2020)^34^	Egypt	52 years	Case report	Tunning test Audiometry tympanometry imaging	SNHL	Unilateral sudden severe to profound SNHL
Jacob et al (2020)^35^	Australia	61 years	Case report		SNHL	Hearing loss
Karimi-Galougahi et al (2020)^36^	Iran	6	Case series	Audiometry	SNHL	Unilateral moderate to severe SNHL
Swain and Pani (2020)^37^	India	472	Prospective study	Audiometry Tympanometry tuning fork tests TEOAEs	SNHL	28 presented with HL, 17 of whom were SSNHL
Thrane et al (2020)^1^	Denmark	225	Retrospective study	Questionnaire	SNHL	24 (10.7%) Hearing Loss
Jin et al (2020)^38^	China		Retrospective study		SNHL	COVID-19 may lead to an increase in patients with SSNHL
Chari et al (2020)^39^	USA	681	Retrospective study		SNHL	13 patients (1.91%) were diagnosed with SSNHL.
Ozer and Alkan (2021)^7^	Turkey	62 years	Case report	PTA, ABR	SNHL	Hearing loss
Degen et al (2020)^14^	Germany	60 years	Case report	ABR	Bilaterally SNHL	Hearing loss
Beckers et al (2021)^40^	Belgium	53 years	Case report	PTA, VHIT	SNHL	Hearing loss
Gerstacker et al (2021)^41^	Germany	38 years	Case report	PTA, TFT, OAE, ABR, VNG		Hearing loss
Guigou et al (2021)^42^	France	29 years	Case report	PTA	SNHL	Bilaterally HL
Pokharel et al (2021)^43^	Nepal	27 years	Case report	PTA, TFT	SNHL	Hearing loss
Edwards et al (2021)^44^	UK	68 years	Case report	PTA	SNHL	Hearing loss
Ricciardiello et al (2021)^45^	Italy	5	Case report	PTA, tympanometry, HST, ABR, THI, DHI	SNHL	Hearing loss
Lang et al (2020)^25^	Ireland	30 years	Case report	PTA	SNHL	Hearing loss
Shah et al (2021)^46^	UK	4	Case series	PTA, tympanometry	SNHL	Hearing loss

SSNHL, sudden sensorineural hearing loss; SNHL, sensorineural hearing loss; CHL, conductive hearing loss; HL, hearing loss, OAE, otoacoustic emission; TEOAE, transient evoked otoacoustic emission; VEMP, vestibular evoked myogenic potential; PTA, pure tone audiometry; ABR, auditory brainstem response; VHIT, video head impulse test; TFT, tunning fork test; VNG, videonystagmography; THI, tinnitus handicap inventory; DHI, dizziness handicap inventory; HST, hearing scale test.

## Results

###  Search results

 A total of 90 papers were retrieved from the initial search strategy. After the first review of papers, 40 duplicates were identified and removed, and the titles and abstracts of the remaining 50 articles were reviewed by researchers, leading to the extraction of 40 studies for full-text assessment. Next, the remaining studies were evaluated in the full-text screening step by applying the eligibility criteria, and finally, 35 studies met the inclusion criteria and were included in the final review. These 35 articles were written in the form of case reports and case series from 2020 to 2022.The included studies investigated the impacts of COVID-19 on the auditory system, within which 96 patients infected with COVID-19 and with hearing loss confirmed by conducting hearing tests were included. Among 96 patients, 21 cases (21.88%) had a complete recovery of hearing loss and 31 cases (32.3%) experienced a partial recovery. Steroid drugs were prescribed for 62 cases (64.59%), and for the rest of the cases, steroid drugs were not prescribed or were not reported. The general characteristics of the reviewed articles are given in Table 1.

###  Neurological symptoms in COVID-19 

 Neurological symptoms of COVID-19 have been reported in more than 80% of severe cases, which could be related to the neurotropic and neuroinvasive properties of the virus.^2^ Neurological symptoms include headache, anosmia, impaired consciousness, delirium and/or encephalopathy, restlessness, stroke, meningoencephalitis, depression, and sleep problems. Analysis of the SARS-CoV-2 genome has shown that this emerging virus shares approximately 80% to 90% sequence identity with the original SARS-CoV. Both bioinformatic modeling and laboratory experiments show that 2019-nCoV uses ACE2 as a receptor to enter human cells. Also, the studies suggest that 24 hours after SARS-CoV infection, the expression of ACE2 increases dramatically compared to the infection at 12 hours. After 48 hours, ACE2 expression remains at a high level. This suggests that ACE2 not only plays an important role in viral susceptibility but may also play a role in post-infectious regulation. Grijalva-Otero^47^ has pointed out to the potential neurotrophic and neurological effects of SARSCoV-2, reporting that one-third of the patients in their study experienced neurological symptoms during COVID-19. The mechanism of central nervous system (CNS) symptoms caused by SARS CoV-2 virus is not fully understood; One of the potential mechanisms of virus entry into the CNS is through the nose near the olfactory epithelium; Thus, research suggests that anosmia and aging may be indicators of virus neuroinvasion.^6,48^

 Recent research has reported significant increased incidence of neurological diseases associated with COVID-19, including Guillain-Barré syndrome, encephalopathy, and stroke. In an Italian group, Guillain-Barré syndrome has been reported in approximately 0.5% of the patients with COVID-19, and the first symptom was flaccid paralysis after the onset of acute respiratory symptoms, but research on this matter is very limited and cannot be definitively commented on.^7^

 Other effects of SARS-CoV-2 on the nervous tissue is the direct CNS infection or vascular injury, which can be attributed to vasculopathy, that is similar to the described infectious mechanism for varicella zoster virus and human immunodeficiency virus (HIV). The latter can be supported by COVID-19 patients showing signs of coagulopathy. Considering the damages caused by this virus in the peripheral system and CNS, damage to the peripheral and central vestibular auditory system is not out of mind.^49^ As a result, in addition to the common clinical manifestations, the presence of neuro-auditory symptoms, such as tinnitus, balance disorders and sudden sensorineural hearing loss (SSNHL), has been reported in COVID-19 patients.^6^

###  Viral infections and the auditory system

 Hearing loss is classified as CHL, SNHL or mixed hearing loss (MHL). CHL is the result of damage to the outer or middle ear that manifests with the transmission disturbance of sound to the inner ear. SNHL is the result of impairment of cochlear, auditory nerve, central auditory perception, or processing function caused by a viral, autoimmune, or idiopathic cause.^50^Viral infections can cause congenital or acquired hearing damage, unilateral or bilateral, which can be mild, severe, or profound. The pathophysiology of viral damage to the auditory system is different. Some viruses can directly damage inner ear structures, including the organ of Corti (home to auditory sensory receptors) and hair cells. Others cause inflammatory responses that damage the structure of the ear. Some also increase sensitivity to bacterial or fungal infections by weakening the immune system, which leads to hearing loss, such as HIV causing transient hearing loss through secondary bacterial infection and suppressing the immune system. Viral hearing loss can be conductive, sensorineural or mixed, but SSNHL is more common, and is generally caused by damage to the structures inside the cochlea (some viruses can also affect the auditory part of the brainstem).^9,49,50^

###  Auditory system and COVID-19 (etiopathology)

 Viral infections may involve cranial nerves, leading to SSNHL, peripheral facial palsy, or disturbances of smell and taste. The etiological factor for SSNHL, caused by COVID-19, shows many similarities with different viruses such as herpes simplex, HIV, hepatitis, measles, rubella, mumps, Lassa virus and enteroviruses. Three mechanisms are involved in the occurrence of SSNHL associated with viral infections: neuritis caused by viral involvement of the cochlear nerves, viral involvement of the cochlea and surrounding lymphatic tissues, and the stress response caused by internal cross-reaction of ear antigens for viral infections.^23^ In addition to all of the mentioned mechanisms, ototoxic drugs such as azithromycin and hydroxychloroquine, which are widely used during the current epidemic, may also play a role in hearing loss and/or balance disorders associated with COVID-19 infection as therapeutic drugs.^8^ Animal studies have reported virally induced hearing loss due to direct involvement of the inner ear or indirectly through the cerebrospinal fluid.^45^

 Histopathological studies^51,52^ of patients with SSNHL have identified the loss of hair cells and supporting cells of the organ of Corti without inflammatory cell infiltration. This suggests that the pathology of idiopathic SSNHL may be related to cellular stress pathways. Also, in COVID-19 infection, nasopharyngeal inflammation and edema may block the Eustachian tube. Obstruction of the Eustachian tube leads to indentation of the tympanic membrane with ear fullness symptom and ear disorders.^12,51^ Also dysfunction of the Eustachian tube may lead to acute otitis media, causing severe ear disorders and hearing loss.^51^

 In a systematic review conducted by Fancello et al,^53^ 15 case reports and case series for a total of 20 patients with SARS-CoV-2 infection was identified by searching databases. All patients reported new onset of vestibular hearing symptoms. The mean age of the patients was 42.4 ± 35 years and the ratio of gender (male vs. female) was 13:7. The recorded time interval between SARS-CoV-2 infection and the onset of vestibular hearing symptoms was a maximum period of 6 weeks. The brain MRI was performed for 10 out of 20 patients with vestibular hearing disorders. Neuroimaging showed a clear inflammatory process of the inner ear in two cases, one bilaterally; this supports the hypothesis of direct damage to the cochlea and labyrinth.^53^

 Although the exact mechanism of damage to the auditory system mechanism by COVID-19 is unknown, some possible mechanisms derived from various research studies includes the followings:(1) *Cochleitis or neuritis* caused by viral involvement of the inner ear or vestibular nerve, potentially leading to vertigo, tinnitus, and hearing loss.^54^(2) *Cross-reactions*: Antibodies or T cells may misidentify inner ear antigens as viruses, leading to accidental damage to the inner ear.^54^ (3) *Vascular disorders*: the cochlea and semicircular canals have no collateral blood, which means they are highly susceptible to ischemia.^55^ Several cardiovascular manifestations, including coagulation abnormalities, have been reported in COVID-19 patients.^56^The consequences of such manifestations may lead to thrombosis or hypoxia of the inner ear.^54^ Micro thrombosis is one of the multifactorial processes that play a role in the development of vascular, malignant and neuroinflammatory diseases and organ failure.^57^ Vascular smooth muscles contain ACE2, thus the virus can infect them and eventually infect the veins that feed the auditory center, creating a new clot in these vessels, or displacing an existing clot. This clot can block the vessels feeding the auditory center and cause ischemic damage. In fact, due to the disorder in the vascular structure and the elderly patient’s susceptibility to thrombosis, auditory problems may occur with the mechanisms mentioned above.^5,40,57^ (4) *Immune reactions*: consequences of immune-mediated disturbances (e.g. excessive production of pro-inflammatory cytokines) may negatively affect the auditory vestibular system.^54^ (5) *Oxygenation of auditory tissue*: the inner ear is an energetic organ. Microvascular networks, which are vital for hearing, are in different places in the cochlea. The main vascular network is in the lateral wall of the cochlea, receiving 80% of the blood flow of the cochlea. The next largest vascular network locating in the bony spiral lamina, are two bony plates from which the vocal cords pass through, and the spiral medulla, which contains spiral ganglion neurons, and receives 19%-24% of blood.^58^ Disturbance in cochlear blood flow is considered as one of the factors involved in the pathophysiology of sensorineural hearing loss types.^59^ When the virus infects red blood cells, it deoxygenates them; thus if there is excessive activation of the virus in the auditory center in the brain, the auditory center may remain hypoxic and damaged.^5,57^

## Discussion

 In this study, we aimed to conduct a review of current evidence that examined hearing loss as a complication of COVID-19. We also examined that whether COVID-19 leads to an increased incidence of hearing loss by using the available literature. SNHL, tinnitus, and/or vertigo have been shown to occur during and following COVID-19 infection.^2^ As COVID-19 pandemic varied across countries, and the inconsistency and even contradictory of the findings, it remains questionable whether COVID-19 contributes to the high incidence of hearing loss.

 Since the outbreak of COVID-19, many atypical symptoms have been reported accordingly. Even though the primary site of SARS-CoV-2 is the lung, it can cause various extrapulmonary symptoms, for example, sensory and neural complications such as sudden onset olfactory and gustatory dysfunction, otologic signs (e.g., tinnitus, vertigo, and hearing loss), nonspecific symptoms, and long-term neurological complications.^60^

 The studies in the present review applied various techniques to confirm that the patient had hearing loss. The tests used to verify hearing loss included pure tone audiometry (PTA), and otoacoustic emissions. PTA helps determine the type and severity of HL.^61^ Some studies have confirmed that it is crucial to establish a clear temporal link between SNHL onset and a confirmed SARS-CoV-2 infection (at the PCR for the rhino-pharyngeal swab).^2^ Despite the small number of studies, the possibility of a relationship between COVID-19 and hearing loss is recently gaining prominence in the scientific literature. The first case mentioning sensorineural hearing loss in a SARS-CoV-2 positive patient was by Sriwijitalai and Wiwanitkit in April 2020.^62^ The pathophysiology of viral damage to the auditory system is different. Some viruses can directly damage inner ear structures and some of them can damage the external ear structures.^12,49,63^ For instance, in a case report, an elderly woman who recovered from a severe COVID-19 infection, referred with sensorineural hearing loss. This has prompted speculation that COVID-19 can increase oxidative stress and promote acute thrombosis, which can cause irreversible damage to the auditory CNS.^62^On the other hand, Fidan observed unilateral CHL with otalgia and tinnitus.^12^ In addition, COVID-19 can have other symptoms such as psychological symptoms or even tinnitus. For example, Manchaiah et al reported that 4 of their 7 patients suffered from tinnitus, impaired sleep and attention dysfunction.^64^

 Most of prior studies theorized that COVID-19 may be a cause of SSNHL, but some studies do not follow this pattern. For example, Chari et al^32^ believe that there is no significant relationship between COVID-19 and hearing loss. They also added that COVID-19 does not appear to confer a significantly increased risk of developing SSNHL, unless infected patients are selectively less willing to risk visiting an emergency service facility in a major hospital system during the acute phase of a pandemic.^32^

###  Conductive hearing loss caused by COVID-19

 There is no evidence of SARS-CoV-2 presence in the middle ear and mastoid, but the presence of other respiratory viruses has been detected in middle ear effusions. Considering that the epithelium of the middle ear and mastoid is like the mucosa of the upper respiratory tract, the middle ear and mastoid may be an important reservoir for viral particles in patients with COVID-19.^65^ Viruses can be the sole infectious agent of acute otitis media or play a co-infection role with bacteria and rarely with other viruses. Viral infections can impair immune function and reduce the natural clearance of mucosal cells by altering the properties of mucosal cells. Nasopharyngeal mucosa and Eustachian tube, which leads to negative middle ear pressure, makes the middle ear prone to the formation of effusion and secondary bacterial or viral infection. Angiotensin-converting enzyme receptor 2 is the cellular entry pathway for SARS-CoV-2. High levels of ACE2 are expressed in the goblet cells of the nasal, basal, and ciliated respiratory epithelium. Because of the enriched population of ciliated cells, glands, and goblet cells in the lower part of the eustachian tube, research suggests that this part of the tube could be a potential route for SARS-CoV-2 to cause otitis media.^17^ Study that was conducted on the tissues collected from the middle ear and nasal septum, 3 hours after the death of a person infected with COVID-19 or during ear, nose, and throat surgeries, identified that the expression of SARS-CoV-2 RNA in the middle ear, and nasal cavity are generally increased in individuals with COVID-19 compared to control tissue obtained from uninfected patients. A study conducted on tissues collected from the middle ear and nasal septum, 3 hours after the death of a person infected with COVID-19 or during ear, nose and throat surgeries, showed that the expression of RNA of COVID-19 in the ear Median and nasal cavity are generally increased in people with COVID-19 compared to control tissue obtained from uninfected patients.^66^ Among the 8 cases that Raad et al^17^ reported, six patients had ear symptoms and seven cases had hearing loss. For six patients, middle ear effusion was evident in the otoscopy examination. Three patients had typical symptoms of acute otitis media, one had acute otitis media with tympanic membrane perforation, and two had olfactory disorders. Interestingly, in one patient, polymerase chain reaction test was positive for middle ear effusion and negative for oropharyngeal swab.^17^ Fidan reported a 35-year-old female patient with tinnitus and ear symptoms, but with no symptom of COVID-19 and no comorbidities. In the ear, nose and throat examination, there was hyperemia and bulging of the tympanic membrane, CHL was observed in the audiometry. There was also bilateral lung involvement on chest X-ray and positive RT-PCR result for COVID-19. After the treatment, it was found that the result of the PCR test was negative, and the chest radiography was normal.^12^

###  Sensorineural hearing loss caused by COVID-19

 The first report on a case of sensorineural hearing loss in a SARS-CoV-2 positive patient was conducted by Sriwijitalai and Wiwanitkit in April 2020.^15^ A meta-analysis showed that the rate of hearing loss in patients with COVID-19 was 3.1%.^67^ A systematic review on all patients undergoing PTA conducted by Fancello et al, showed unilateral SNHL in 36 patients and bilateral SNHL in 27 patients. Hearing loss ranged from mild to moderate in 58.7%, from moderate to severe in 4.8%, and from severe to profound in 36.5% of the patients. None of the patients reported complete hearing improvement. Complete SNHL treatment rate was reported to be12.5%.​​^2^ Dror et al reported that the asymptomatic SNHL patients with COVID-19 infection, had significantly worse hearing thresholds at frequencies of 4, 6, and 8 kHz, and lower mean TEOAE (Transient Evoked Otoacoustic Emission) compared to matched controls.^68^

 In a study conducted in 2022 by Bozdemir et al, a total of 24 patients with mild/moderate COVID-19 and 24 healthy individuals with the matched age and sex were compared. The average pure tone of the patients was significantly higher than the control group; this difference was due to the higher average pure tone of the COVID-19 patients at 4 kHz. However, there was no significant difference between frequency-specific pure tones of patients and the control group. The amplitude of TEOAEs in the patients was significantly lower than the control group at the frequency of 4 kHz and 5 kHz. In their study, normal tympanometry results showed that the middle ear was spared from COVID-19 infection. According to these findings, it can be said that COVID-19 may lead to damage of outer hair cells, which are mainly located in the basal turn of the cochlea, and the hearing problems associated with this disease might be related to the cochlea rather than the central auditory connections.^69^

###  Sudden hearing loss caused by COVID-19

 SSNHL is defined as sensorineural hearing loss of 30 db. or more, in at least three consecutive frequencies occurring over 72 hours.^15,70^ Many studies have proven the connection between COVID-19 and SSNHL. In a systematic review, a direct link between COVID-19 and the onset of SSNHL was concluded.^71^ Sadiq et al,^50^ also, reported a case of sudden MHL, which was one of the few reported cases with concurrent SNHL and CHL. He reported a 68-year-old woman with positive PCR test. In the ear evaluation, retraction of the tympanic membrane with a slight decrease in mobility, and no lesion or effusion was observed. In the audiometric evaluation, moderate bilateral pure tone MHL, with the left side being more affected was diagnosed. The patient reported a gradual improvement of HL over time, and announced improvement approximately 2 months after the onset of SARS-CoV-2 infection.^51^ These findings suggest that MHL can be a marker for SARS-CoV-2, even in mild to moderate manifestations.

 Wong reported a 35-year-old man, with positive PCR test for COVID-19, who referred with sudden hearing loss in the left ear, accompanied by vertigo and fullness in the left ear. In otoscopic examination, healthy tympanic membranes were observed. His tympanometry was also normal; but the PTA test showed unilateral sensorineural hearing loss in the left ear. Subsequently, his hearing improved clinically. He also reported another 45-year-old man with COVID-19. On day 20 of post-COVID-19, he referred with a new onset of left ear symptoms, dizziness, and vomiting. Bilateral tympanogram and PTA showed moderate to severe left sensorineural hearing loss. Little clinical/audiological improvement was observed after 2 months.^72^

 In a 2021 systematic review,^54^ the prevalence of hearing loss was reported to be approximately 8%, within which SNHL was the most common type. Among all 56 studies included in their review, 30 (54%) reported hearing loss and 29 (52%) reported existence of the symptom. Nine case/series studies and a cross-sectional study reported SSNHL that was relatively similar in pattern to typical SSNHL. In these latter cases, SARS-CoV-2 may not be the only cause of SSNHL. However, it does not preclude the possibility that SARS-CoV-2 causes SSNHL.^54^

###  Auditory neuropathy

 It has also been reported that some neurological symptoms such as peripheral neuropathy, encephalitis, and Guillain–Barre syndrome (GBS) may develop in the long term (up to three weeks after the onset of respiratory symptoms of COVID-19).^73^ Acquired auditory neuropathies (AAN) are a group of hearing disorders identified by abnormal auditory conduction despite normal cochlear function.^74^ The mechanisms reported to cause neuropathy due to viral infection was either an indirect mechanism via an antibody response that would cross-react with an inner ear antigen, or a direct mechanism due to invasion of the cochlear nerve or anterior labyrinth.^42^ A recent report describes a possible causal relationship between COVID-19 and GBS, suggesting that COVID-19 can cause sensorineural hearing loss through auditory nerve dysfunction, such as AAN.^75,76^ For example, in a 29-year-old male case diagnosed with SARS-CoV-2 who presented with sudden onset of bilateral hearing loss, moderate bilateral sensorineural hearing loss was diagnosed. Cochleitis with bilateral auditory neuropathy and the signs of endolymphatic fluid inflammation were observed in his MRI results.^42^ Another potential mechanism for AAN among patients with COVID-19 infection is the development of ischemic brain infarction or hemorrhage.^68^ It is likely that symptomatic COVID-19 infections may alter neuronal auditory brainstem response (ABR) signals and prolong inter-peak wave latencies. If the auditory pathways are involved, it may be associated with AAN, although this theory needs to be investigated in a cohort of patients with severe symptomatic COVID-19 infection.^68^

###  Sudden SNHL treatment

 One of the important implications of identifying COVID-19 in the etiology of SSNHL is to choose the appropriate treatment strategy to maximize clinical improvement and minimize side effects. Reduced-dose of oral prednisolone and B vitamins with folic acid complex are beneficial in SNHL in COVID-19 patients, and corticosteroids also play a key role in the treatment of SSNHL.^23^However, for COVID-19 infection, as in many other viral infections, the use of corticosteroids might cause the risk of increasing the severity of the infection and delaying the clearance of the virus.^77^Investigating the presence of SARS-CoV-2 in patients with SSNHL complaints and using other alternative treatment methods in positive cases of COVID-19 can prevent such adverse outcomes.^23^ A study showed that the symptoms of 56.25% of COVID-19 patients who had hearing loss, became normal with the use of corticosteroids.^72^ In non-COVID-19 patients, the recovery rate from SNHL after treatment with corticosteroids in the first week of disease onset, within 2 weeks and after 3 months was 87%, 52% and less than 10%, respectively.^8^

 In the diagnosis and treatment of ISSNHL, the results suggest that earlier treatment with steroids is better than later treatment, which follows the accepted practice guidelines for ISSNHL.^78^Early diagnosis and treatment with steroids, and referral by primary care providers to an otolaryngologist can facilitate combination therapy of oral steroids and high-dose IT (intratympanic). Age of onset may potentially affect outcomes, but combined oral and parenteral administration may not.^78^

## Limitation

 In this study we examined the relationship between the onset of hearing loss and the infection of COVID-19 by reviewing research in this area. We found that different studies used varying methods to assess hearing loss making it challenging to establish a correlation between the two. Some studies relied on self-reported symptoms instead of conducting examinations and objective tests which may have led to underreporting. Additionally, we discovered that the occurrence of hearing loss is not limited to the symptoms of COVID-19 but can manifest during recovery. Therefore, it is crucial to monitor individuals with confirmed cases of COVID-19 both at the time of infection and throughout their recovery process. It’s important to note that due to the circumstances brought about by the overwhelmed hospitals many people, with COVID-19 choose not to undergo follow up hearing assessments.

 Given the significance of exploring how this virus impacts hearing loss it is crucial to carry out research with a large number of patients. It is essential to employ methods and adhere, to the COVID-19 standard along with an extended follow up period. Moreover, early treatment for hearing loss hearing loss is strongly advised. Consequently, it is recommended to establish policies for hearing evaluation and screening, in individuals diagnosed with COVID-19.

## Conclusion

 Hearing loss in COVID-19 infection has not received much attention by health care professionals. COVID-19 infection could have deleterious effects on the inner ear specifically on the hair cells of the cochlea, despite patients being asymptomatic. SNHL, tinnitus, and/or vertigo have been shown to occur during and following COVID-19 infection. As COVID-19 pandemic varied across countries, and the inconsistency and even contradictory of the findings, it remains questionable whether COVID-19 contributes to the high incidence of hearing loss. SSNHL is a non-specific symptom in COVID-19 infection. However, it can be the sole symptom in COVID-19 infection which helps to recognize the positive case. Awareness of this nonspecific symptom may be targeted in this pandemic for prevention of the infections by isolating the patient. Early identification of SSNHL in COVID-19 patients can save the hearing of the patient. The proper understanding of the mechanisms behind hearing loss in COVID-19 infections needs further research. Nonetheless, it seems likely that SNHL could be included among the manifestations of those-called “long COVID” syndrome. Our findings in this review may assist health practitioners, audiologists, healthcare providers, and health decision-makers to have a better understanding of possible pathologies of the disease and its association with hearing loss, with the hope to determine appropriate medical treatment plans.

## Competing of Interests

 The authors declare that they have no competing interest.

## Ethical Approval

 This study was approved by Ethical Committee of Tabriz University of Medical Sciences, Ethical Number: IR.TBZMED.VCR.REC.1401.435.
